# Willingness to use remote patient monitoring among cardiovascular patients in a resource-limited setting: a cross-sectional study

**DOI:** 10.3389/fdgth.2024.1437134

**Published:** 2024-09-17

**Authors:** Mitiku Kassaw, Getasew Amare, Kegnie Shitu, Binyam Tilahun, Bayou Tilahun Assaye

**Affiliations:** ^1^ Department of Health Informatics, College of Medicine and Health Science, Debre Markos University, Debre Markos, Ethiopia; ^2^ Department of Health System and Policy, Institute of Public Health, College of Medicine and Health Science, University of Gondar, Gondar, Ethiopia; ^3^ Department of Health Education and Behavioral Sciences, Institute of Public Health, College of Medicine and Health Science, University of Gondar, Gondar, Ethiopia; ^4^ Department of Health Informatics, Institute of Public Health, College of Medicine and Health Science, University of Gondar, Gondar, Ethiopia

**Keywords:** cardiovascular disease, remote patient monitoring, non-communicable diseases, willingness, Ethiopia

## Abstract

**Introduction:**

Currently, mortality by non-communicable diseases is increasing alarmingly. They account for approximately 35 million deaths each year, of which 14% are due to cardiovascular disease and 9.2% occur in Africa. Patients do not have access to healthcare services outside the healthcare setting, resulting in missed follow-ups and appointments and adverse outcomes. This study aimed to assess the willingness to use remote monitoring among cardiovascular patients in a resource-limited setting in Ethiopia.

**Method:**

An institution-based cross-sectional study was conducted from April to June 2021 among cardiovascular patients at referral hospitals in Ethiopia. A structured interview questionnaire was used to collect the data. A systematic random sampling technique was used to select 397 study participants. Binary and multivariable logistic regression analyses were employed and a 95% confidence level with a *p*-value <0.05 was used to determine the level of association between variables.

**Result:**

In total, 81.61% of the study participants were willing to use remote patient monitoring [95% confidence interval (CI) = 77.4%–85.1%]. Age [adjusted odds ratio (AOR) = 0.94; 95% CI: 0.90–0.98], having a mobile phone (AOR = 5.70; 95% CI: 1.86–17.22), and perceived usefulness (AOR = 1.50; 95% CI: 1.18–1.82) were significantly associated with willingness to use remote patient monitoring among cardiovascular patients.

**Conclusion:**

Cardiovascular patients had a high willingness to use remote patient monitoring. Age, perceived usefulness of remote patient monitoring, and having a mobile phone were significantly associated with a willingness to use remote patient monitoring.

## Introduction

In the 21st century, mortality due to non-communicable diseases (NCDs) has been increasing alarmingly and it has been the leading threat to human health and development (1). NCDs account for approximately 35 million deaths each year, of which 14% were due to cardiovascular diseases (CVDs) (2) and 9.2% of the total mortality occurred in Africa (3).

CVDs are a cluster of disorders of the heart and blood vessels that include but are not limited to ischemic heart disease (IHD), heart failure, rheumatic heart disease (RHD), peripheral arterial disease (PAD), and stroke (4) In a study conducted in Ethiopia, more than 3 million people were found to affected by CVDs. Of these cases, 33.7% were RHD, followed by IHD (22.5%) and stroke (11.4%) (5).

NCDs accounted for 34% of deaths in Ethiopia in 2011, 31% in 2014, and 39% in 2018. Of these, CVDs contributed 15% to NCD-related deaths in 2011, 9% in 2014, and 16% in 2018.

In addition to this, the age-standardized CVD prevalence was 5,534 per 100,000 population in Ethiopia (6, 7). A study conducted in Tikur Anbessa referral hospital showed that 70% of RHD affects mainly the working-age group (18–40 years) (8).

According to the World Health Organization (WHO), the healthcare system is experiencing a rapid digital transformation to deliver healthcare services remotely using broadcast communications innovation (8) through an automated web, electronic, or phone-based information transmission of diagnosis, treatment of diseases, injuries, and physiological data such as heart rate, blood pressure, oxygen saturation, and weight where healthcare providers and patients are spatially separated (9–11), thus using audio, video, and other telecommunication technologies to monitor vital parameters of patient status (9).

In developed countries, remote patient monitoring (RPM) has been used to monitor the health status of patients outside the healthcare setting and as an alternative approach to reduce geographical barriers and optimize healthcare service access where in-person visits are difficult. Evidence shows that remote monitoring allows for better communication between the patient and the physician for timely treatment (12, 13). Remote patient monitoring benefits the patients with immediate feedback at the earliest sign of health treatment (14).

However, implementing remote patient monitoring in resource-limited settings can present several challenges, such as a lack of the necessary infrastructure and technology, a shortage of financial resources, ensuring patient data privacy and security, and training healthcare professionals to monitor and interpret patient data remotely (15). Successful RPM implementation relies on patient engagement and adherence to monitoring protocols, where patients may have limited health literacy or cultural beliefs that affect their willingness to participate in RPM programs (16).

Integrating RPM into existing healthcare systems can be complex, particularly in resource-limited settings where the healthcare infrastructure may be fragmented or underdeveloped for interoperability, and data integration with electronic health records (EHRs) may pose challenges (17). Addressing these challenges requires a multi-faceted approach involving collaboration between healthcare providers, technology vendors, policymakers, community stakeholders, and patients (18). It is critical to adapt RPM solutions to the unique context of resource-limited settings, taking into account the available resources, infrastructure, and cultural factors (14, 19, 20).

An RPM system reduces the healthcare personnel burden and medico-legal issues (13, 21). For instance, mobile phone-based remote monitoring is a relatively cheap and convenient way to improve heart failure patient management (22). Remotely managing the patient using telemedicine has emerged as an alternative to optimize therapy, improve quality of life, prevent readmission, and self-manage their condition (13, 20–22). Wireless remote monitoring allows clinicians to make clinical decisions on time rather than in the office (14). The use of remote monitoring reduces hospitalization by 44%and prevents secondary cause mortality (23). In the USA, RPM for patients with cardiovascular heart failure (CHF) was cost-effective (24), reducing the need for hospitalization and mortality (22, 25).

Tele-monitoring in the Netherlands of patients with COVID-19 enabled them to recover in their homes, improved satisfaction, reduced hospitalization, and reduced healthcare costs (13). Efficiency and satisfaction resulted from remote monitoring of patients in the COVID-19 watch program at the University of Pennsylvania (26). In sub-Saharan Africa (SSA), tele-health interventions in the diagnosis, tracking, and care of patients with COVID-19 were effective (27) and remote monitoring of patients with human immunodeficiency virus (HIV) and tuberculosis (TB) contributed to the reduction of healthcare costs, waiting time, hospital visits, and improved patients’ quality of life (22).

Patients are constrained by unnecessary transportation, long waiting times, and healthcare costs (27, 28). These challenges to providing health services in rural and urban communities in Ethiopia are due to limited health facilities and health personnel (29). The WHO emphasized designing different models to manage NCD (30).

The Ethiopian Ministry of Health (MoH) has set key strategic directions for digital health technology to enhance the healthcare system, such as strengthening health information systems (HIS), implementing EHR, and telemedicine and remote healthcare (28). However, there is limited evidence on the acceptance and willingness of cardiovascular (CV) patients to use RPM services. Therefore, this study aimed to assess the willingness to use remote monitoring and associated factors among cardiovascular patients in a resource-limited setting.

## Method and materials

### Study design and setting

An institutional-based cross-sectional study was conducted among 423 cardiovascular patients. The study was conducted at specialized teaching hospitals in the Amhara region from April to June 2021. These hospitals have been used as teaching and referral centers for a population of more than 10 million in the catchment area.

### Inclusion and exclusion criteria

Patients who were 18 and older and had a follow-up in the hospitals during data collection were included in the study. However, patients who were seriously ill and unable to respond were excluded from the study.

### Operational definitions

#### Cardiovascular disease

Patients with cardiac disease (ischemic, rheumatic, chronic heart failure), stroke, PAD, or deep vein thrombosis (DVT).

#### Willingness to use remote patient monitoring

A patient who responded “yes” was considered to be willing to use remote monitoring for their disease management, and those who responded “no” were unwilling to use remote monitoring (31).

#### Attitude to use remote patient monitoring

A 5-point Likert scale was used ranging from “strongly disagree” to “strongly agree” with eight items and classified into favorable and unfavorable attitudes using Bloom's cut-off point. An attitude score of less than 79% (<32 points) was grouped into an unfavorable attitude, while a score of greater than 80% (32–40 points) was considered a favorable attitude toward using remote monitoring (32).

#### Perceived usefulness

Perceived usefulness was measured using a 5-point Likert scale ranging from “strongly disagree” to “strongly agree” by three items. A higher usefulness score implied a patient perceived remote monitoring to be more useful as compared to a patient with a lower usefulness score (33).

#### Perceived ease of use

Perceived ease of use was measured using a 5-point Likert scale that ranged from “strongly disagree” to “strongly agree” by four items. A higher ease of use score meant a patient perceived remote monitoring to be easy to use (34, 35).

### Sample size and sampling procedures

The sample size was determined using the single population proportion formula by considering the 95% confidence interval (CI), 5% marginal error (d = 0.05), and 50% of the magnitude of willingness to use remote patient monitoring since there has been no previous study done in the same population among CV patients (*p* = 0.5). After accounting for a 10% non-response rate, the total final sample size was 423. A systematic random sampling technique was used to select the study participants. The expected number of patients during the data collection period was 917, which was obtained from two referral hospitals. An interval was calculated as k = N/n, where N is the expected number of CV patients during the data collection period and n is the sample of CV patients (423), thus k = 917/423 = 2. Thus with an interval of 2, CV patients were selected based on their order of registration and this continued until the sample number was reached.

### Data collection tools and procedures

A structured administrative questionnaire was adapted and modified from various literature studies to collect data including socio-demographic characteristics, attitude toward RPM, technological factors, clinical factors, healthcare access, and willingness to use remote monitoring (35–37). The content validity of the questionnaire was checked, and the reliability was calculated using Cronbach's alpha coefficient (=0.70), which was acceptable. The questionnaire was first prepared in English and then translated into the local Amharic language and back into English by experts to ensure consistency.

### Data quality control

A pre-test was conducted outside the actual study sites among 10% of CV patients; before the actual data collection, modifications were made based on the pre-test. Two interns and two bachelor of science (BSC) nurses participated as data collectors and supervisors, respectively. The data collectors and the supervisor were trained before participating in the actual data collection process. To create awareness of the purpose of the study, their rights, and confidentiality issues, sufficient time was given to respondents to read and fill in materials carefully. There was continuous supervision up to the end of data collection. After collecting the data, the supervisor and the investigator checked its consistency and completeness.

### Data management and analysis

The data were collected using the KoboCollect tool and then checked, cleaned, and exported into Excel to reduce error and incompleteness. The data was exported and analyzed using STATA 14. The descriptive statistics results were expressed as mean, standard deviation, percentage, and frequency. Binary logistic regression was employed to identify factors associated with RPM use. Variables with a *p*-value ≤0.2 from the bi-variable analysis were considered during multivariable analysis. The multivariable logistic regression analysis was used to control potential confounders and identify significant factors associated with willingness to use RPM. The magnitude of the association between different independent variables and dependent variables was measured using adjusted odds ratios (AORs). The Hosmer–Lemeshow goodness of fit was used to test the model's fitness. Multi-collinearity between independent variables was assessed by checking their tolerance and variable inflation factors (VIF). A 95% CI and a *p*-value <0.05 were used to declare statistical significance.

## Results

### Socio-demographic characteristics

In total, 81.61% of the study participants had a willingness to use remote patient monitoring (95% CI = 77.4%–85.1%). A total of 397 cardiovascular patients participated in the study, with a response rate of 94%. The mean age of the study participants was 49 ± 13.19 years. More than half (51.64%) of the participants were males, and approximately 65% (258) were from urban areas. One-third of (31.7%) the participants had not received any formal education (Table 1).

**Table 1:** Socio-demographic characteristics of cardiovascular patients in the referral hospitals, 2021 (*n* = 397).

Variables	Frequency	%
Sex		
Male	205	51.64
Female	192	48.36
Residence		
Urban	258	64.99
Rural	139	35.01
Religion		
Christian	273	68.77
Muslim	124	31.23
Marital status		
Single	49	12.34
Married	348	87.66
Educational status		
No formal education	126	31.74
Primary (1st–8th)	62	15.62
Secondary (9th–12th)	81	20.40
College and above	128	32.24
Employment		
Unemployed	111	27.96
Employed	286	72.04
Payment method		
Health insurance	198	49.87
Poverty card	11	2.77
Out-of-pocket	188	47.36
Age^a^ [mean (SD)]	49.26 (±13.19)	
Monthly income^a^ [median (IQR)]	127.59 (±113.92)	

^a^ Continuous.

### Clinical characteristics of participants

In total, 55.92% of the study participants were classified as cardiac and 16.12% were hypertensive. More than half of the total participants (51.89%) were found to have comorbidities, of which 56.31% of the patients were found to have hypertension, 93.7% of patients were taking their medication orally, and 59.00% of the patients missed their follow-up due to COVID-19 (Table 2).

**Table 2:** Clinical characteristics of cardiovascular patients in the referral hospitals in 2021 (*n* = 397).

Variables	Frequency	%
Main diagnosis for follow-up		
Cardiac	222	55.92
Hypertension	64	16.12
Stroke	56	14.11
PAD	32	8.06
DVT	23	5.79
Route of medication		
Orally	372	93.70
Injection	25	6.30
Comorbidity		
Yes	206	51.89
No	191	48.11
Type of comorbidity (*n* = 206)		
Hypertension	116	56.31
DM	35	7.77
Stroke	25	12.14
Cardiac	16	17.00
Others^a^	44	6.31
Missed follow-up		
No	175	44.08
Yes	222	55.92
Reason to miss follow-up (*n* = 222)		
I forgot it	121	54.50
I was sick	91	40.10
Distance	17	7.65
Others^b^	16	7.20
Due to COVID-19	131	59.00
Miss medication		
No	175	44.08
Yes	222	55.92
Reason to miss medication (*n* = 222)		
I was away from home	171	77.02
Many pills	53	23.87
I was confused about the dosage	32	14.41
People told me the medication was not good	47	21.17
Due to money	21	9.45
Frequency of follow-up		
Every 2–3 week	39	9.82
Monthly	236	59.45
Every 2–3 months	122	30.73
Duration of illness^c^ **[**median (IQR)]	3 (±3)	

^a^ HIV+ and TB+.

^b^ Lack of money.

^c^ Continuous.

### Technological factors

Of the total study participants, 80.86% were mobile phone users of which 52.03% were smartphone users (Table 3).

**Table 3:** Technological factors of cardiovascular patients in the referral hospitals, 2021 (*n* = 397).

Variables	Frequency	%	
Mobile phone			
No	76	19.14	
Yes	321	80.86	
Smartphone (*n* = 321)			
No	154	47.97	
Yes	167	52.03	
Use smartphone apps (*n* = 167)			
No	7	4.19	
Yes	160	95.81	
Download smartphone apps (*n* = 167)			
No	36	21.55	
Yes	131	78.55	
Computer access			
No	252		63.48
Yes	145		36.52
Computer skill			
No	223		60.27
Yes	147		39.73
Internet use			
No	219		55.30
Yes	177		44.70
Frequency of Internet use (*n* = 177)			
Weekly	11		6.21
On most days of the week	62		35.03
Daily	104		58.76
RM 1st choice			
Voice call	196		49.37
Video call	177		44.58
Text messaging	7		1.76
Customized smart device (e.g., wireless blood pressure monitoring device, etc.)	17		4.28
Perceived usefulness^a^ [median (IQR)]	13.00 (±4.00)		
Perceived ease of use^a^ [mean (SD)]	14.63 (±1.71)		

^a^ Continuous.

### Healthcare access

Of the total participants, 63.73% reported that traveling from home to the hospital was convenient, and of the study participants, 62.97% reported a bus as their mode of transport. Of the participants, the median traveling time was 1.5 h from home to the hospital to access healthcare services, and patients were waiting for more than 2 h during their follow-up day (Table 4).

**Table 4:** Healthcare access factors of cardiovascular patients in the referral hospitals, 2021 (*n* = 397).

Variables	Frequency	%
Travel from home to hospital convenience		
No	144	36.27
Yes	253	63.73
Mode of transport		
Walking	21	5.29
By Bus	250	62.97
Walking and by bus	126	31.74
Someone accompanies me for an appointment today		
No	118	29.72
Yes	279	70.28
Time taking from home to hospital [median (IQR)]	1.50 (±1.50)	
Waiting time for an appointment today [median (IQR)]	2.30 (±1.00)	

### Factors associated with willingness to use remote patient monitoring

The study participant's age, perceived usefulness of RPM, and use of a mobile phone were significant factors associated with willingness to use remote patient monitoring among cardiovascular patients.

A year increase in the age of the patients decreased the odds of being willing to use remote monitoring by 6% (AOR = 0.94, 95% CI: 0.90–0.98). Cardiovascular patients who had mobile phones were five times more likely to use remote monitoring (AOR = 5.00 95% CI: 1.65–14.55). For a unit increase in a cardiovascular patient’s score of perceived usefulness of RPM, the odds of being willing to use remote monitoring increased by a factor of 1.5 (AOR = 1.50, 95% CI: 1.16–1.76) (Table 5).

**Table 5:** Bi-variable multivariable logistic regression analysis of factors with the willingness to use remote monitoring among cardiovascular patients in the referral hospitals, 2021 (*n* = 397).

Variables	Willing to use RPM		Crude odd ration (COR) (95% CI)	AOR (95% CI)
	Yes (%)	No (%)		
Sex				
Male	173 (84.40)	32 (15.61)	1.46 (0.88–2.44)	0.80 (0.34–1.88)
Female	151 (78.65)	41 (21.40)	1	1
Residence				
Urban	233 (90.31)	25 (9.70)	4.91 (2.86–8.44)	2.20 (0.68–7.08)
Rural	91 (65.47)	48 (34.53)	1	1
Religion				
Christian	218 (79.85)	55 (20.15)	0.67 (0.37–1.20)	1.23 (0.52–2.93)
Muslim	106 (85.50)	18 (14.50)	1	1
Educational status				
No formal education	83 (65.87)	43 (34.13)	1	1
Primary (1st–8th)	48 (77.42)	14 (22.58)	1.77 (0.88–3.57)	0.36 (0.12–1.07)
Secondary (9th–12th)	72 (88.89)	9 (11.11)	4.14 (1.89–9.08)	0.41 (0.11–1.53)
College and above	121 (81.61)	7 (5.47)	8.95 (3.84–20.87)	0.63 (0.16–2.42)
Missed follow-up				
No	158 (90.80)	16 (9.20)	1	1
Yes	165 (74.32)	57 (25.68)	0.3 (0.16–0.52)	0.36 (0.12–1.04)
Ever missed medication				
No	158 (90.80)	16 (9.20)	1	1
Yes	165 (74.32)	57 (25.68)	0.26 (0.14–0.48)	0.67 (0.24–1.85)
Mobile phone				
No	32 (42.11)	44 (57.89)	1	1
Yes	292 (90.97)	29 (9.03)	13.84 (7.64–25.07)	5.00 (1.65–14.55)*
Attitude				
Favorable	291 (84.59)	53 (15.41)	3.32 (1.77–6.23)	1.43 (0.57–3.56)
Unfavorable	33 (62.26)	20 (37.74)	1	1
Traveling convenience				
No	102 (70.83)	42 (29.17)	1	1
Yes	222 (87.75)	31 (12.25)	2.94 (1.75–4.95)	1.13 (0.47–2.74)
Someone accompanies				
No	107 (90.68)	11 (9.32)	1	1
Yes	217 (77.78)	62 (22.22)	0.35 (0.18–0.71)	1.20 (0.45–3.15)
Age (years)^a^	47.14 (±12.74)	58.70 (±10.89)	0.92 (0.90–0.94)	0.94 (0.90–0.98)**
Monthly income^a^	129.55 (±70.88)	114.47 (±71.99)	1.00 (0.99–1.00)	0.99 (0.99–1.00)
Duration of illness^a^	3.54 (±2.25)	5.36 (±3.08)	0.77 (0.69–0.84)	0.91 (0.79–1.05)
Perceived usefulness^a^	12.72 (±1.96)	10.90 (±2.52)	1.46 (1.28–1.65)	1.50 (1.16–1.76)**
Ease of use^a^	14.87 (±1.54)	13.56 (±1.97)	1.64 (1.37–1.95)	1.13 (0.91–1.41)
Time taken from home to hospital^a^ (h)	1.83 (±1.00)	2.14 (±1.07)	0.76 (0.60–0.95)	1.21 (0.81–1.80)

^a^ Continuous.

* *p* < 0.001; **p < 0.05.

## Discussion

Telemedicine has made significant strides in improving patient-to-provider communication, expanding access to previously underserved populations and places, and improving the delivery of healthcare. The study participant's age, the perceived usefulness of RPM, and the use of a mobile phone were significant factors associated with willingness to use remote patient monitoring among cardiovascular patients.

The study showed that 81.61% of cardiovascular patients were willing to use remote monitoring for their follow-up. This finding was in line with studies conducted in Honduras (38), Santiago (39), and Poland (40). However, this figure was higher than the findings from Nigeria (40), Singapore (38), and Canada (41). The discrepancies might be due to differences in socio-demographic variations. For instance, in the study conducted in Singapore, the participants were patients with diabetes mellitus type 2 (DM-2), whereas in our study, the participants were cardiovascular patients. In the study from Nigeria, the educational status of the participants was lower than that of the participants in our study. As for the difference in study setting, the studies mentioned above were conducted in primary care clinics, whereas the current study was done at teaching hospitals. Moreover, study period variation may be another possible reason for the observed discrepancies since technologies are improving over time (35, 38), which explains the increased use of remote monitoring in the current study compared to studies done in previous years. However, the current finding is lower than a study done in the USA (42). This variation might be due to patients’ experience with technology in the USA being higher than the current study’s participants (43).

Age, perceived usefulness of RPM, and having a mobile phone were significantly associated factors with the willingness to use remote patient monitoring. Our study showed that the odds of using remote monitoring decreased by 6% in response to a year increase in the age of the patients. A patient's willingness to use remote monitoring decreases as age increases. A possible reason for this might be that older patients are less willing to use technology than younger patients (due to lower intention toward technology, anxiety to use, concern about the security of information, and cost) which was evident in studies conducted in Germany (44, 45), Australia (46), China (47), and Canada (22).

The current finding showed that cardiovascular patients who had mobile phones were five times more likely to use remote monitoring. This finding is supported by studies done in Australia (46), China (47), Greece (48), and Kenya (49). We found that more than two-thirds of cardiovascular patients had mobile phones. The widespread use of mobile phones suggests an increase in the tendency to use remote monitoring (50). Currently, mobile phones are the preferable way to communicate with healthcare providers directly via text messaging or voice calls regardless of where they are located. This implies that patients may be willing to use remote monitoring for their health service demands if those services are launched.

Ethiopia has struggled to implement digital health for managing chronic disease to improve the health of society and effectively facilitate the delivery of healthcare services (51, 52). The current study showed that cardiovascular patients with higher perceived usefulness of RPM were 1.5 times more likely willing to use remote monitoring. However, scholars have shown that there are Internet connectivity issues or data transmission errors, privacy concerns, patient compliance issues, and the need for healthcare professionals to interpret and act upon the data effectively (53). Clinically, the data obtained through remote monitoring can be utilized in various ways. It can provide valuable insights into a patient's health status, allowing for early detection of potential issues, monitoring treatment effectiveness, and facilitating personalized healthcare management, which can aid in making informed clinical decisions and designing individualized interventions (54).

Different types of healthcare providers may follow such data, depending on the specific condition being monitored. It can be used by primary care physicians, specialists, nurses, and other healthcare professionals involved in the patient's care (55). The influence of remote monitoring data on interventions can be significant. It enables healthcare providers to intervene promptly and make timely adjustments to treatment plans based on real-time information. For example, if abnormal vital signs are detected, healthcare providers can initiate the appropriate interventions (56).

This finding is supported by studies conducted in Singapore (36), Sweden (57), and Taiwan (32), where patients’ willingness to use remote monitoring was found to be associated with their perception of the usefulness of the RPM technology. This may be because if patients perceive remote monitoring as useful, they have a propensity to use remote monitoring to manage their disease conditions to improve their health. The perceived usefulness of remote monitoring indicates one's acceptance and the perceived utility of remote monitoring and this will have a positive influence on a patient's willingness to use RPM (33).

Digital infrastructure, including strong network connectivity, and the development of different digital platforms to effectively collect and process data from cardiac patients to manage their healthcare conditions, will be crucial for evidence-based planning and decision-making (58, 59).

## Conclusion

The study participants had a high willingness to use remote patient monitoring to maintain their health and quality of life and showed a substantial willingness to use remote patient monitoring. Age, the perceived usefulness of remote monitoring, and the use of a mobile phone were significantly associated with a willingness to use remote monitoring among cardiovascular patients.

This enabling finding should inspire policymakers, particularly the Ministry of Health, to enhance and broaden the use of remote monitoring technologies in healthcare services by encouraging cardiovascular patients to utilize remote patient monitoring technology for disease management.

## Data Availability

The raw data supporting the conclusions of this article will be made available by the authors, without undue reservation.
